# Synthesis and Characterization of Curcumin-Polycaprolactone Block Copolymers for Biomedical Applications

**DOI:** 10.3390/ma18184348

**Published:** 2025-09-17

**Authors:** Qianqian Wei, Adam Junka, Bartlomiej Dudek, Houman Alimoradi, Julia Simińska-Stanny, Lei Nie, Oseweuba Valentine Okoro, Armin Shavandi

**Affiliations:** 1 Université libre de Bruxelles (ULB), École Polytechnique de Bruxelles-BioMatter, Avenue F.D. Roosevelt, 50-CP 165/61, 1050 Brussels, Belgium; qianqian.wei@ulb.be (Q.W.); houman.alimoradi@ulb.be (H.A.); julia.siminskastanny@ulb.be (J.S.-S.); oseweuba.okoro@ulb.be (O.V.O.); 2 Platform for Unique Models Application PUMA, Department of Pharmaceutical Microbiology and Parasitology, Wroclaw Medical University, Borowska 211, 50-556 Wroclaw, Poland; adam.junka@umw.edu.pl (A.J.); bartlomiej.dudek@umw.edu.pl (B.D.); 3 College of Life Sciences, Xinyang Normal University, Xinyang 464000, China

**Keywords:** curcumin, polycaprolactone, block copolymer, electrospinning

## Abstract

Polycaprolactone (PCL) is widely utilized in biomedical applications such as tissue engineering and drug delivery; however, its limited bioactivity remains a key challenge. In this study, bioactive curcumin–polycaprolactone block copolymers (MCP) were synthesized via ring-opening polymerization of ε-caprolactone and maleic anhydride modified curcumin. The resulting MCP was characterized using FTIR, ^1^H NMR, UV–Vis spectroscopy, and differential scanning calorimetry (DSC). It demonstrated enhanced antioxidant activity, UV-blocking capacity, and electro spinnability compared to PCL. Electrospun MCP films exhibited improved biocompatibility and promoted fibroblast migration. Furthermore, composite films incorporating MCP into a PVA matrix with and without copper or iron were evaluated for in vivo toxicity and antimicrobial activity. These formulations showed no systemic or contact toxicity in the *Galleria mellonella* model, confirming their biocompatibility. Films containing copper or iron exhibited selective anti-*Pseudomonas aeruginosa* activity and low but reproducible antioxidant capacity. This study highlights the multifunctionality and biomedical potential of MCP and its composites as tunable platforms for regenerative and antimicrobial applications.

## 1. Introduction

Biomaterials play a crucial role in regenerative medicine, drug delivery, and medical implants via their use in the fabrication of functional scaffolds, characterized by enhanced biocompatibility and bioactivity which promotes controlled interactions with biological systems. Natural biomaterials, such as collagen, gelatin, and alginate, provide biocompatibility and bioactivity, promoting cell adhesion, proliferation and tissue regeneration. However, they often exhibit weak mechanical properties, limited processability, inadequate stability, high batch-to-batch variability, and challenges in functionalization [1,2,3]. Synthetic polymers including poly (ε-caprolactone) (PCL), polylactic acid (PLA), polyvinyl alcohol (PVA), and polyethylene glycol (PEG) offer higher tunability in mechanical strength, degradation rate, and structural design compared to natural biomaterials [4,5,6]. However, they often exhibit limited bioactivity and biocompatibility, necessitating further modifications to enhance their biological performance [7]. Therefore, the development of biomaterials with enhanced properties that can be widely applied in the biomedical field remains an ongoing challenge.

Among synthetic polymers, poly(ε-caprolactone) (PCL) offers an advantageous combination of biocompatibility, biodegradability, and cost-effectiveness, along with desirable properties such as flexibility, tunable mechanical strength, and excellent processability. These characteristics enable its fabrication into various forms applied in biomedical fields, including fibers, scaffolds, films, and microspheres, through advanced techniques such as electrospinning, 3D printing, and solvent casting. Notably, electrospun PCL nanofibers, with their high surface area-to-volume ratios and structural similarity to the extracellular matrix, effectively support cell proliferation, differentiation and adhesion, making them widely applied in skin tissue engineering, nerve regeneration, wound healing, bone repair, and biosensors.

In addition, PCL has gained significant attention in the field of drug delivery. Its controlled degradability and biocompatibility make it an ideal carrier for sustained and localized drug release. For example, PCL-based systems have been employed to deliver vancomycin for osteomyelitis, providing controlled release and promoting bone healing [8]. Multilayer PCL/chitosan coatings with drug-loaded microspheres have demonstrated controlled antibiotic release [9]. These examples highlight PCL’s versatility for designing effective and controlled-release delivery systems.

Nevertheless, the application of PCL remains limited due to its inherent hydrophobic nature and low bioactivity. To address these shortcomings, various functionalization strategies have been developed to enhance PCL’s biological performance. These include direct copolymerization of tannic acid with PCL [10], synthesizing PCL-poly (ethylene glycol) (PEG) multiblock copolymers [11], and grafting PCL onto macromolecules such as lignin [12]. Additionally, incorporating bioactive agents such as growth factors, peptides, ceramics, or natural compounds (e.g., curcumin), into PCL based biomaterials, has been explored to improve cell proliferation, collagen synthesis, angiogenesis, and tissue repair.

Among these bioactive agents, curcumin, a natural polyphenol extracted from the rhizome of *Curcuma longa* (turmeric), has been widely studied for its low toxicity, high biocompatibility, and multifunctional properties, including anti-inflammatory, anti-infective, antioxidant, antibacterial, metal-chelating, and radiosensitizing effects [13,14,15,16]. For example, Liu et al. developed a polyaniline–curcumin composite with strong antimicrobial activity and accelerated wound healing [14]. Boschetto et al. showed that curcumin improved the bioactivity of bioinert acrylic resin, promoting bone regeneration [17]. Furthermore, curcumin chelates metals to form metal-curcumin complexes, which have been reported to exhibit enhanced bioavailability [18]. For example, Barik et al. [19] demonstrated that the coordination of curcumin with copper significantly enhanced its free radical scavenging activity compared to curcumin alone. Bhattacharjee et al. [20] showed that a zinc-curcumin complex applied to fluoride doped hydroxyapatite exhibited enhanced antibacterial efficacy, osteosarcoma inhibition, and osteoblast growth, benefiting bone tissue engineering and dental applications.

Despite its promising bioactivity, the biomedical application of curcumin is hindered by its hydrophobicity, chemical instability, poor bioavailability, and rapid systemic clearance, limiting its clinical translation. To overcome these challenges, various curcumin-loading strategies have been developed, including lipid-based systems, polymeric nanoparticles, solid lipid nanoparticles, hydrogels, and porous carriers. However, these approaches often face limitations such as complex fabrication processes, suboptimal loading efficiency, and curcumin degradation during storage or delivery [21,22].

This study investigated the synthesis of a bioactive block co-polycaprolactone via ring-opening polymerization of ε-caprolactone and curcumin, incorporating maleic anhydride (MA) into the backbone. Furthermore, the processability of the maleic anhydride-modified curcumin–PCL (MCP) copolymer was evaluated using electrospinning, 3D printing and molding techniques for the fabrication of fibers, scaffolds and microchannels, demonstrating its versatility for diverse biomedical applications.

## 2. Material and Methods

### 2.1. Materials

The monomer ε-caprolactone, tin (II) 2-ethylhexanoate (Sn (Oct)_2_)-Toluene solution (0.1 g/mL), tetrahydrofuran (THF), curcumin (Cur), and maleic anhydride (MA), dimethyl sulfoxide (DMSO) were obtained from Tokyo Chemical Industry (TCI) Co., Ltd. (Tokyo, Japan). Dichloromethane (DCM), sodium hydroxide, diethyl ether (purity: 99.5%), methanol (purity: 99.8%), were bought from Chemical-Lab Co., Ltd. (Taito-ku, Tokyo, Japan). Phosphate-buffered saline (PBS), polyvinyl alcohol (PVA), and 1,1-diphenyl-2-picrylhydrazyls (DPPH) were purchased from Sigma-Aldrich (Millipore Sigma) (St. Louis, MO, USA). Iron (III) chloride and copper (II) chloride dihydrate were supplied by Merck KGaA (Darmstadt, Germany). Tetrahydrofuran-D (purity: 99.5%, TDF) and chloroform-D (purity: 99.5%) (CDCl_3_) were bought from Eurisotop (Saint-Aubin, Île-de-France, France). ABS-like resin ink was purchased from Shenzhen Anycubic Technology Co., Ltd. (Shenzhen, China). 1% penicillin–streptomycin, Dulbecco’s modified Eagle’s medium (DMEM) with high glucose, trypsin, fetal bovine serum (FBS), Alamar Blue (AB), and 3-(4,5-dimethylthiazol-2-yl)-5-(3-carboxymethoxyphenyl)-2-(4-sulfophenyl)-2H-tetrazolium (MTS) were purchased from Thermo Fisher Scientific (Waltham, MA, USA). The fibroblast 3T3-L1 transformed cell line was supplied by the ATCC^®^ (American Type Culture Collection) (Manassas, VA, USA). All reagents were used as received without further purification.

### 2.2. Preparation of Maleic Anhydride Modified Curcumin (MC)

**Scheme 1:**
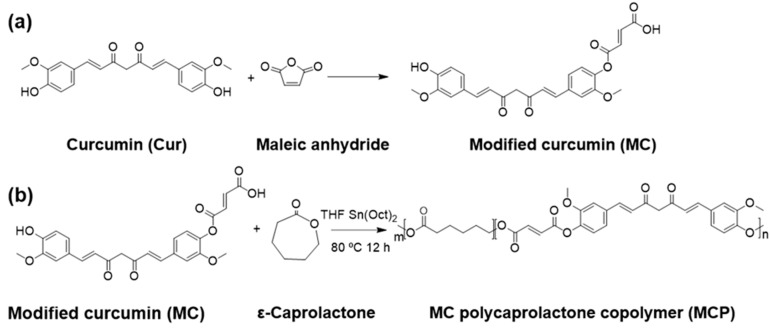
Synthetic schemes for (**a**) maleic anhydride modified curcumin (MC) and (**b**) MC polycaprolactone copolymer (MCP).

Maleic anhydride modified curcumin (MC) was synthesized by reacting maleic anhydride and curcumin at a 1:1 molar ratio [23] (Scheme 1a). Briefly, 1.0 g of curcumin and 0.27 g of maleic anhydride were separately dissolved in 10 mL of THF each, under stirring. The 10 mL of maleic anhydride solution was then added dropwise to the curcumin solution, and the reaction was allowed to take place for 12 h at reflux (65 °C). The resulting solution was dried under vacuum at 40 °C using a rotary evaporator (Rotavapor^®^ R-100-Buchi, Büchi Labortechnik AG, Flawil, Switzerland) to obtain the maleic anhydride modified curcumin product.

### 2.3. Preparation of Maleic Anhydride Modified Curcumin–PCL (MCP)

The MCP was synthesized via ring-opening polymerization (ROP) of ε-caprolactone [10,24,25] (Scheme 1b). A 50 mL dried glass reaction bottle was initially degassed and purged with nitrogen (N_2_). Then, 1.0 g of MC was dissolved in 10 mL of THF, followed by the sequential addition of 4 mL of caprolactone monomer and 0.4 mL of 0.1 g/mL Sn(Oct)_2_-Tolune solution as the catalyst. The sealed bottle was immersed in an oil bath at 80 °C and maintained under reflux with nitrogen protection for 12 h. The reaction mixture was then cooled and dissolved in dichloromethane (DCM), followed by precipitation using excess iced cold diethyl ether. This dissolve-and-precipitate process was repeated twice. The resulting MCP was dried under vacuum (850 mbar) at 40 °C using a rotary evaporator until constant weight was achieved and then subjected to further characterization.

### 2.4. Preparation of MCP-Metal Complexes

MCP metal complexes were prepared following a previously reported method with minor modifications [26]. Briefly, 1 mmol of metal salt was dissolved in 5 mL of methanol at room temperature (25 °C). Separately, 3.0 g of MCP copolymer was dissolved in 40 mL of methanol and sonicated in an ultrasonic bath until complete dissolution was achieved. The metal salt solution was then added dropwise to the MCP copolymer solution under constant stirring. After 20 min of stirring, a solution of NaOH (40 mg) in 1.0 mL MeOH (prepared immediately prior to use) was added dropwise to adjust the pH (to ~5 for the Cu salt solution). On the other hand, for the MCP and Fe complex, the pH of the solution was adjusted to ~3.0 using a dilute aqueous HCl solution (0.1 M), added dropwise under stirring. The reaction mixture was subsequently stirred for 1 h. Upon completion of the reaction, the product was collected by precipitation with cold diethyl ether and separated by centrifugation. The solid was washed repeatedly with methanol to remove unbound metal salts and then dried under reduced pressure using a rotary evaporator. The purified products were denoted MCP–Fe and MCP–Cu, respectively.

### 2.5. Electrospinning of PCL, MCP and MCP-Based Composite

The bioactive block copolymer of curcumin and PCL, and the MCP-based composite, was electrospun (SKE E-Fiber EF100, SKE Research Equipment, Vicenza, Italy). Initially, 1 g of PCL was dissolved in 8 mL of THF and poured into a 10 mL syringe. The electrospinning process for PCL was carried out at a flow rate of 0.78 mL/h with a 21-gauge needle (inner diameter is 0.5 mm), a voltage of 15 kV, and a collector speed of 225 rpm at room temperature. Similarly, a 2 g sample of MCP was weighed and fully dissolved in 6 mL of THF to prepare the MCP solution for electrospinning. The electrospinning was performed at a flow rate of 0.03 mL/h, a voltage of 12 kV, and a collector speed of 225 rpm.

In a separate preparation, 2 g of polyvinyl alcohol (PVA) was completely dissolved in 20 mL of deionized water. Then, 5 mL of the resulting solution was loaded into a 21-gauge needle and was electrospun at 12 kV onto a collector surface pre-coated with an aluminum foil layer, with the collector rotating at 225 rpm. Subsequently, for the fabrication of PVA-based composite fibers, 2 g of MCP, MCP-Cu, or MCP-Fe was dissolved in 6 mL of THF to prepare the respective electrospinning solutions. These were electrospun at a flow rate of 0.03 mL/h, a voltage of 12 kV, and a collector speed of 225 rpm. The resulting fibrous mats were designated as PVA, PVA-MCP, PVA-MCP-Cu, and PVA-MCP-Fe, respectively.

### 2.6. Structural Characterization by FTIR and ^1^H NMR

The chemical structures of PCL, curcumin, MC, and the resulting copolymer (MCP) were confirmed by Fourier transform infrared spectroscopy (FTIR) and proton nuclear magnetic resonance (^1^H NMR) spectroscopy. FTIR spectra were recorded using a Bruker Alpha II spectrometer (Bruker Optics GmbH & Co. KG, Ettlingen, Germany) in attenuated total reflectance (ATR) mode, over the range of 4000–500 cm^−1^ with a resolution of 1 cm^−1^ and an average of 32 scans per sample. For ^1^H NMR analysis, Cur, MC, and MCP were dissolved in deuterated solvents at a concentration of 10 mg/mL and transferred into NMR tubes. The spectra were obtained using a 400 MHz JEOL JNM-ECZ400 R/S3 spectrometer (JEOL Ltd., Tokyo, Japan) to verify structural modifications and polymer composition.

### 2.7. Thermal and Spectroscopic Characterization

Differential scanning calorimetry (DSC) was used to evaluate the thermal properties of PCL, MCP, and curcumin. Samples (6–10 mg) were sealed in aluminum crucibles and analyzed under a nitrogen atmosphere using a heating/cooling rate of 5 °C/min. PCL and MCP were scanned from 0 °C to 130 °C, while curcumin underwent two heating–cooling cycles between 0 °C and 200 °C. Ultraviolet–visible (UV–Vis) spectroscopy was performed to analyze the conjugated systems of Cur, MCP, and MCP-metals. Samples were dissolved in DMSO and scanned in the range of 200–800 nm using a PerkinElmer Lambda 25 UV–Vis spectrophotometer (PerkinElmer, Inc., Waltham, MA, USA).

### 2.8. Antioxidant Analysis

The antioxidant activities of Cur, MC, MCP and MCP-based composite electrospun films (PVA, PVA-MCP, PVA-MCP-Cu, PVA-MCP-Fe) were evaluated using the stable free radical 1,1-diphenyl-2-picrylhydrazyl (DPPH). For Cur, MC and MCP, each sample (1 mg/mL) was dissolved in THF and mixed with a 0.25 mM DPPH solution in THF at a 1:1 (*v*/*v*) ratio. For the MCP-based composite electrospun films, 20 mm × 20 mm fragments were immersed in 1.0 mL of 0.1 mM DPPH in methanol in sterile 24-well plates. All mixtures were incubated in the dark for 30 min, and absorbance was subsequently measured at 517 nm using a UV–Vis spectrophotometer. Film blanks (methanol only) and DPPH-only controls were included, and ascorbic acid (100 µg/mL) served as a positive control. DPPH scavenging activity was calculated as follows:

○ For Cur, MC and MCP

(1) $$DPPH\;scavenging\;(\%)=\frac{A_{0}-A_{1}}{A_{0}}\times 100\%$$

where A_0_ and A_1_ represent the absorbances of the control group (DPPH solution without sample) and the sample, respectively.

○ For MCP-based composite electrospun films

(2) $$Scavenging\;Activity\;(\%)=\frac{A_{control}-(A_{sample}-A_{blank})}{A_{control}}\times 100\%.$$

where A_control_, A_sample_ and A_blank_ represent the absorbances of the control (DPPH alone), the sample and the blank (methanol only), respectively.

### 2.9. Scanning Electron Microscope (SEM) Analysis

A field emission scanning electron microscope (SEM, FEI Quanta 200 FEG, FEI, Hillsboro, OR, USA) was employed to evaluate the morphologies, microstructure, and diameter parameters of the dried MCP, PCL electrospinning and MCP electrospinning. Prior to observation, the samples were mounted onto a sample holder and coated with a 15 nm platinum layer to enhance conductivity and mitigate charging effects during imaging.

### 2.10. Biological Evaluation of MCP Electrospun Films

The in vitro biocompatibility and cell migration responses of electrospun MCP and PCL films were evaluated using 3T3-L1 fibroblasts. Cells were cultured in high-glucose DMEM supplemented with 10% fetal bovine serum (FBS) and 1% penicillin–streptomycin under standard conditions (37 °C, 5% CO_2_). Electrospun samples were sterilized via UV exposure (2 h) and incubated in cell culture media (10 mg film per mL medium) for 24 h at 37 °C to prepare extraction solutions. Cells were then exposed to these extracts for up to 72 h to assess cytocompatibility.

#### 2.10.1. Alamar Blue Assay

Cells (8000/well) were seeded in 96-well plates with 300 μL of MCP or PCL extract and incubated for 24, 48, and 72 h. Wells containing medium alone or sample extract without cells served as controls. At each time point, 30 μL of AB reagent was added and incubated for 3 h at 37 °C in the dark. Absorbance at 540 and 630 nm was measured using a microplate reader (Epoch, BioTek^®^, BioTek Instruments, Winooski, VT, USA). AB reduction (%) was calculated using the following formula:

(3) $$AB\;reduction\;(\%)=\frac{\left(\varepsilon_{ox}\;\lambda_{2})(A\lambda_{1})-(\varepsilon_{ox}\;\lambda_{1})(A\lambda_{2}\right)}{\left(\varepsilon_{red}\;\lambda_{1})(A^{\prime }\lambda_{2})-(\varepsilon_{red}\;\lambda_{2})(A^{\prime }\lambda_{1}\right)}\;\;\times 100\%\;$$

where ε_ox_ and ε_red_ denote the molar extinction coefficients of AB at 540 nm (λ_1_) and 630 nm (λ_2_) in the oxidized and reduced forms, and Aλ_1_/λ_2_ and A′λ_1_/λ_2_ represent the absorbances of the test and negative control wells at the corresponding wavelengths.

#### 2.10.2. MTS Assay

In parallel, MTS assays were conducted using the same cell density and extraction conditions. After 24, 48, and 72 h of incubation, 20 μL of MTS reagent was added to each well and incubated for 2 h at 37 °C. Absorbance was then measured at 492 nm to quantify cell metabolic activity.

### 2.11. Scratch Assay for Cell Migration of MCP Electrospun Films

To evaluate the influence of electrospun materials on fibroblast migration, a scratch assay was performed. Cells (1.2 × 10^4^/well) were seeded in 96-well plates and cultured for 24 h. After removing the medium, a linear scratch was introduced using a 10 μL pipette tip, followed by gentle PBS washing to remove detached cells. Fresh PBS was added to prevent drying, and initial images of the scratch area were captured. PBS was then replaced with 100 μL of medium or sample extract (10 mg/mL), and cells were incubated for another 24 h. After incubation, the media was replaced with PBS, and images were taken to assess wound closure and cell migration.

### 2.12. UV Blocking Test of MCP Electrospun Films

The UV-blocking capability of electrospun MCP membranes was evaluated using a cell-based assay with fibroblast 3T3-L1 cells, following a modified protocol [27]. Cells were seeded into 96-well plates at a density of 8000 cells/well and allowed to adhere for 24 h under standard conditions (37 °C, 5% CO_2_). Following cell attachment, different regions of the same plate were covered with aluminum foil, PCL electrospun membrane, or MCP electrospun membrane and carefully positioned directly above the wells to shield the cells from UV exposure without contacting the culture medium. Wells without any covering served as the control group. The plate was then irradiated with UV light (Specific wavelength: 254 nm; intensity of 0.74 mW/cm^2^; dose equivalent: 2.664 J/cm^2^) for 1 h using a UV lamp installed inside a sterile laminar flow cabinet. The distance between the UV source, the membranes, and the cells was carefully maintained at 40 cm to ensure uniform exposure. After exposure, all covers were removed, and the plate was incubated for 24 h under standard culture conditions. Cell morphology was subsequently observed and recorded using brightfield microscopy to assess UV-induced damage and the protective effect of the coverings.

### 2.13. Systemic Toxicity Assessment in Galleria Mellonella Model of MCP-Based Composite Electrospun Films

To evaluate systemic toxicity, sixth-instar *Galleria mellonella* larvae (200 ± 20 mg) were used. MCP-based composite electrospun films (PVA, PVA-MCP, PVA-MCP-Cu, PVA-MCP-Fe) were cut into 10 mm × 10 mm pieces, immersed in 1 mL of PBS, and vortexed for 3 × 1 min. Samples were incubated at 6 °C for 24 h to prepare eluates. After vortexing, 10 µL of each eluate was injected into the last left proleg of each larva (n = 6 per group) using a sterile Hamilton syringe. Control groups received PBS or 25% ethanol. Larvae were incubated at 30 °C in darkness in separate Petri dishes lined with moist filter paper for 168 h. Viability was monitored daily based on body turgor, melanization, mobility, and response to tactile stimuli. Death was defined as complete immobility with melanization and no reaction to stimulation. Each experimental group consisted of 6 larvae (24 per tested sample type (6 × 4), plus 6 larvae treated with ethanol, plus 6 larvae treated with PBS; n = 36 in total).

### 2.14. Contact Toxicity Assay in Galleria Mellonella Model of MCP-Based Composite Electrospun Films

Circular electrospun film sections (90 mm diameter) (PVA, PVA-MCP, PVA-MCP-Cu, PVA-MCP-Fe) were placed on the bottom of sterile Petri dishes. Three *Galleria mellonella* larvae were added to each dish (n = 3/group). Filter paper treated with 1% SDS served as the positive control. Larvae were incubated at 30 °C in darkness and monitored for 168 h for signs of melanization, immobility, and cuticular damage [28].

### 2.15. Modified Disk Diffusion Assay of MCP-Based Composite Electrospun Films

Electrospun films (25 mm × 25 mm) (PVA, PVA-MCP, PVA-MCP-Cu, PVA-MCP-Fe) were placed on Mueller–Hinton agar plates inoculated with *Staphylococcus aureus* ATCC 6538, *Pseudomonas aeruginosa* ATCC 15442, and *Candida albicans* ATCC 10231, adjusted to 0.5 McFarland standard. Plates were incubated at 37 °C for 24 h, and zones of inhibition were imaged and quantified using ImageJ 1.53a.

### 2.16. Antibiofilm Activity Measurement (Modified A.D.A.M. Assay) of MCP-Based Composite Electrospun Films

To evaluate biofilm inhibition, Mueller–Hinton agar (42.5 mL) was poured into 50 mL conical tubes and inoculated with the same microbial strains. After 24 h biofilm formation, film samples (PVA, PVA-MCP, PVA-MCP-Cu, PVA-MCP-Fe) in sterile culture inserts (25 mm diameter) were suspended above the agar. After another 24 h incubation, 1% TTC in Mueller–Hinton agar was added and incubated for 4 h at 37 °C. Formazan was extracted with ethanol/acetic acid (90:10 *v*/*v*) and absorbance was measured at 490 nm. Biofilm survival was calculated relative to untreated controls.

### 2.17. Bacterial Adhesion Assay via TTC Reduction of MCP-Based Composite Electrospun Films

Electrospun films (PVA, PVA-MCP, PVA-MCP-Cu, PVA-MCP-Fe) were placed in Petri dishes and inoculated with 25 µL of microbial suspensions (same strains, 1 McFarland). After 4 h incubation at 37 °C, 5 µL of 1% TTC was added to the droplets and incubated for 3 h. The resulting red formazan was extracted using ethanol/isopropanol/HCl (95:4:1 *v*/*v*/*v*) and absorbance was measured at 490 nm. The viability was normalized to polystyrene surfaces set at 100%.

### 2.18. Fabrication of Maleic Anhydride Modified Curcumin–PCL Scaffolds

Maleic anhydride modified curcumin–PCL scaffolds were fabricated using different processing techniques, including extrusion-based 3D printing and molding, to demonstrate the material’s versatility in forming biomedical structures. Extrusion printing was performed using a GeSIM BioScaffolder 3.2 (Radeberg, Germany). MCP was loaded into a 10 mL metal syringe and heated to 60 °C to reach a molten state. After vortexing for 2 min to eliminate bubbles and ensure homogeneity, the material was extruded through a 400 μm metal nozzle at a speed of 3 mm/s under 80 kPa pressure with the syringe and nozzle maintained at 60 °C. A glass slide was used as the collection substrate and care was taken to ensure proper adhesion during printing. Molds with embedded channels and predefined features were designed in a CAD software and fabricated using Asiga Max X27 DLP printer (Asiga, Sydney, Australia) (27 μm resolution) with ABS-like Resin+. Molten MCP was cast into the molds (at 60 °C) and allowed to cool gradually to solidify at room temperature. After full solidification, the molded scaffolds were demolded, yielding channel-containing constructs with potential for fluidic or perfusion-based biomedical applications.

### 2.19. Statistical Analysis

All experiments were performed in triplicate. Results were expressed as mean ± standard deviation. Statistical analysis was conducted using one-way ANOVA followed by Tukey’s HSD post hoc test. Significance was set at *p* < 0.05. GraphPad Prism 10.42 (GraphPad Software, Solana Beach, CA, USA) was used for all analyses.

## 3. Results and Discussion

### 3.1. Synthesis and Structural Characterization of MC and MCP

**Figure 1:**
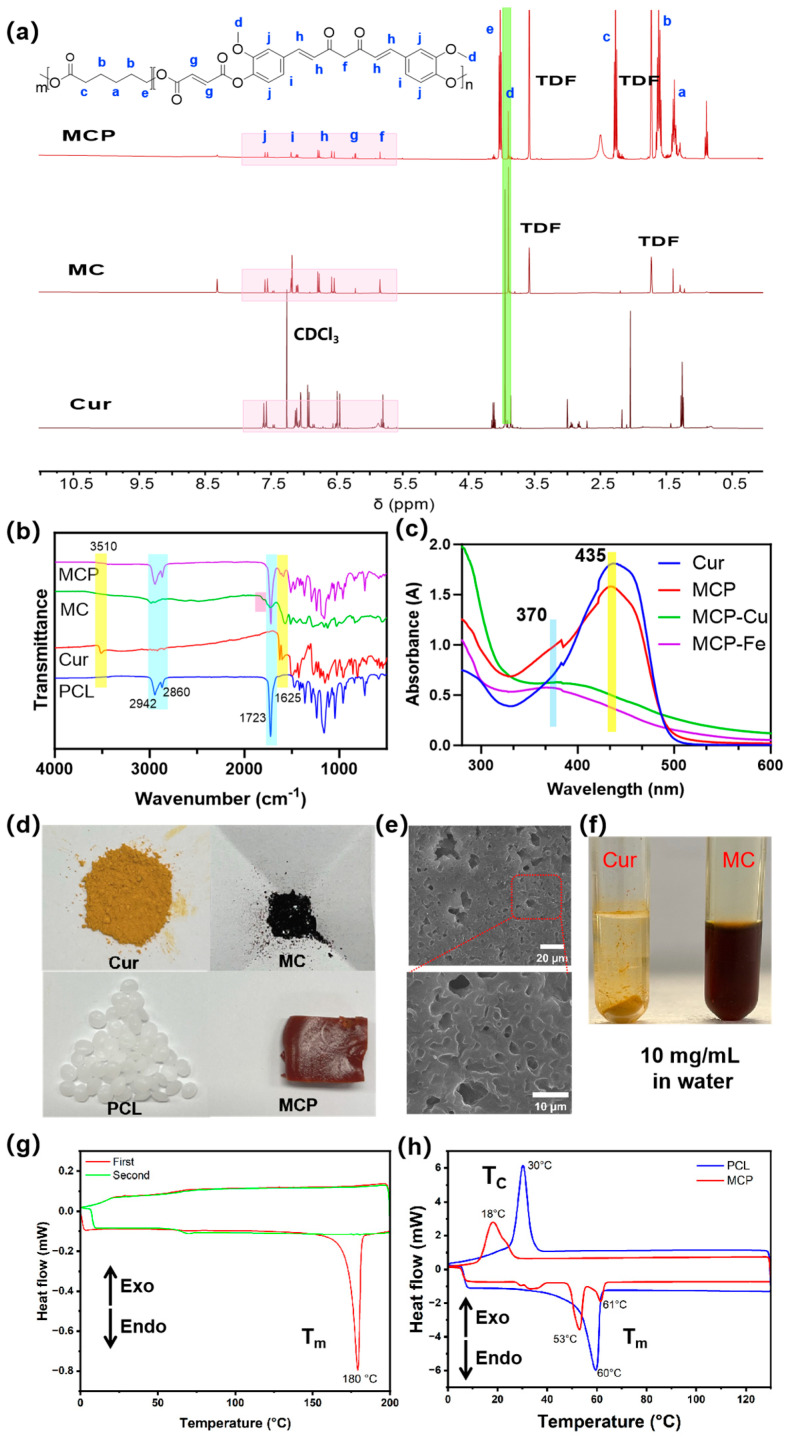
Characteristic properties of the copolymer and its compounds. (**a**) ^1^H NMR results of curcumin (Cur), MC, and MCP. The highlighted sections of green and pink show the methoxy groups and aromatic region signals of curcumin, respectively; (**b**) FTIR of PCL, Cur, MC, and MCP; (**c**) the UV–Vis spectrum of Cur, MCP, MCP-Cu, and MCP-Fe in DMSO solution; (**d**) photographic images of curcumin, MC, PCL, and MCP; (**e**) the SEM images of MCP at 2000× and 6000×; (**f**) hydrophilic properties of curcumin and MC in water with the concentration of 10 mg/mL; (**g**) DSC thermogram of curcumin with two heating and cooling cycles; (**h**) DSC thermogram of PCL and MCP. (Endo: endothermic process; Exo: exothermic process; T_c_: crystallization temperature; T_m_: melting temperature).

Maleic anhydride modified curcumin–PCL requires esterification with maleic anhydride. This transformation was confirmed by NMR and FTIR analyses. The ^1^H NMR spectra of MC and MCP (Figure 1a) exhibit all characteristic signals of curcumin, including the methoxy proton signal at 3.9 ppm, benzylic protons at 5.8 ppm, and the aromatic region (6.6–7.7 ppm) [29,30,31]. Compared to Cur, a new peak at 6.2 ppm appeared in the spectra of MC and MCP, indicating the presence of alkene protons from the maleic acid group. Additionally, a new peak at 8.8 ppm was observed in the MC spectrum, which can be attributed to the hydroxyl group of MC. The MCP spectrum displayed distinct signals characteristic of PCL at 4.0, 2.3, 1.6, and 1.4 ppm [32]. Furthermore, NMR integration results indicate that the ratio of PCL to Cur in MCP is approximately 50:1 (Figure S1).

FT-IR analysis of MC and MCP aligns with the ^1^H NMR spectra. In Figure 1b, Cur displays characteristic peaks, including phenolic O-H stretching at 3510 cm^−1^, aromatic C = O stretching at 1625 cm^−1^ and 1596 cm^−1^, and C = C vibrations at 1504 cm^−1^. MC exhibits additional peaks at 1707 cm^−1^ and 1735 cm^−1^, corresponding to the C = O stretching of the ester and acid groups in its structure [33,34]. The phenolic O-H peak at 3510 cm^−1^ is prominent in Cur and MC but significantly weaker in MCP, indicating consumption of the free phenolic O-H groups of Cur during copolymer formation. MCP retains the aromatic C = C bonds of Cur at 1625 cm^−1^ and 1596 cm^−1^ [35,36,37], alongside characteristic PCL signals including asymmetric and symmetric CH_2_ vibrations at 2942 cm^−1^ and 2860 cm^−1^, and ester carbonyl stretching at 1723 cm^−1^ [38], confirming the presence of both Cur and PCL in the copolymer structure.

Additionally, MCP appeared as red block-shaped solids with smooth surfaces, contrasting the yellow powder of curcumin, crystalline black MC, and blocky white PCL (Figure 1d). MC showed enhanced water-solubility than Cur under 10 mg/mL concentration (Figure 1f). SEM analysis (Figure 1e) revealed that MCP exhibited an interconnected porous structure with a smooth and homogenous surface, free of visible inorganic residues [39]. The presence of MA likely promoted esterification between anhydride groups and terminal hydroxyls of PCL chains, reducing phase separation and resulting in a uniform copolymer morphology [40,41].

### 3.2. Spectroscopic and Thermal Characterization of MCP

UV–Vis spectroscopy was employed to verify the incorporation of curcumin into the MCP copolymer. As shown in Figure 1c, both MCP and curcumin displayed a strong absorption peak at 435 nm, attributed to a low-energy π–π* transition associated with the conjugated chromophore of curcumin [42,43]. This result further confirms the presence of curcumin moieties in the MCP structure. In contrast, MCP-Cu and MCP-Fe exhibited a low-intensity peak at 370 nm, indicating the formation of curcumin-metal complexes [44,45]. The proposed chelation mechanism is illustrated in Figure S2.

Differential scanning calorimetry (DSC) was used to assess the thermal behavior of curcumin, PCL, and MCP (Figure 1g,h). In the first heating cycle, curcumin showed a sharp endothermic peak at 180 °C, corresponding to the melting point of crystalline curcumin However, no thermal transitions were observed in the second cycle, suggesting that curcumin decomposes upon heating rather than undergoing melting–recrystallization. For PCL, a single melting peak was observed at 59 °C, along with a recrystallization peak at 30 °C. In contrast, MCP exhibited two distinct endothermic peaks at 53 °C and 61 °C, and a lower recrystallization temperature of 18 °C. These thermal shifts indicate that the incorporation of maleic anhydride modified curcumin into the PCL matrix reduces crystallinity and alters thermal stability [46,47]. The disrupted crystallization behavior is consistent with the formation of a copolymer with modified packing and thermal response.

### 3.3. Morphologies of MCP Electrospun Fibers

**Figure 2:**
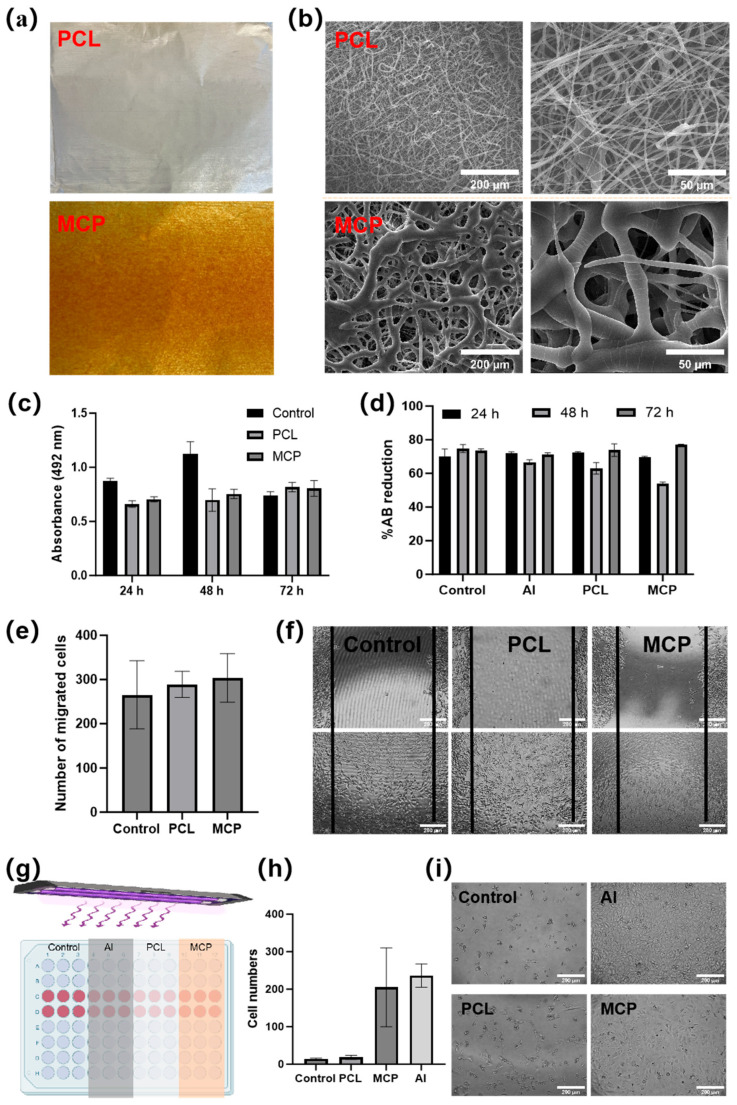
The surface morphology and bioactivities of PCL and MCP electrospinning. (**a**) The optical of PCL and MCP electrospinning; (**b**) the SEM images of PCL and MCP electrospinning; (**c**) the biocompatibilities of PCL and MCP electrospinning evaluated by MTS at 492 nm; (**d**) the biocompatibilities of PCL and MCP electrospinning evaluated by Alamar Blue (AB); (**e**) the number of migration cells; (**f**) cell migration abilities after co-culture with PCL and MCP electrospinning extracts for 24 h; (**g**) the graph of UV-blocking experiments; (**h**) the cell numbers after UV-blocking test with the normal shape; (**i**) the cell morphology after UV light and protection by aluminum foil, PCL, and MCP fiber mats for 24 h.

The morphology of electrospun MCP and PCL films was evaluated by optical and scanning electron microscopy (SEM). As shown in Figure 2a, the MCP films exhibit a distinct yellow color due to the presence of curcumin, in contrast to the colorless appearance of PCL films. SEM images (Figure 2b) reveal that MCP films possess a more interconnected and heterogeneous fibrous architecture compared to the uniform, smooth fibers observed in PCL films. The average fiber diameter of PCL films was 1.03 ± 0.38 µm, whereas MCP films exhibited larger fibers with a diameter of 21.8 ± 5.3 µm. These differences suggest that incorporation of MC alters both the structural organization and fiber formation during the electrospinning process. Such an increase in fiber size is likely attributable to changes in solution viscosity and/or electrical conductivity, which can strongly influence jet stability and fiber thinning.

### 3.4. Biocompatibility and Cell Migration of MCP Electrospun Fibers

The biocompatibility of PCL and MCP electrospun films was assessed using MTS and Alamar Blue (AB) assays over 24, 48, and 72 h of incubation (Figure 2c,d). In the MTS assay, absorbance at 492 nm increased over time for both films, indicating cell proliferation. Although the control group showed higher absorbance values, a slight decrease after 48 and 72 h was observed, likely due to over confluency and nutrient depletion. The AB assay further confirmed that cell viability was maintained across all groups throughout the 72-h period, supporting the non-cytotoxic nature of both PCL and MCP films.

To evaluate the effect of MCP on cell motility, a scratch assay was performed using 3T3-L1 fibroblast cells exposed to extracts from the electrospun films. As shown in Figure 2e, both PCL and MCP extracts supported cell migration after 24 h, surpassing the control. By 24 h, scratch closure was evident in both film-treated groups (Figure 2f), indicating that MCP not only supports biocompatibility but also promotes fibroblast migration, an essential feature for tissue regeneration applications.

### 3.5. UV-Block Capability of MCP Electrospun Fibers

Ultraviolet (UV) radiation is known to induce excessive production of reactive oxygen species (ROS), trigger inflammatory responses, and cause DNA and ocular damage, ultimately contributing to photoaging [48]. Curcumin has been reported to provide UV protection by absorbing UV light through its conjugated π-electron system, carbon–carbon double bonds, and carbonyl functional groups [49,50,51]. To evaluate the UV-blocking ability of MCP electrospun films, cells were exposed to UV light under different protective conditions (Figure 2g). In the control group, cells were directly exposed to UV light without any covering. Other groups were shielded using aluminum foil, PCL, or MCP electrospun membranes. As shown in Figure 2h, cells in the control group displayed reduced viability and abnormal morphology. Figure 2i further illustrates the spherical, non-aggregated morphology of these UV-exposed cells, indicating damage and impaired growth. Cells shielded by PCL showed partial protection, with some cytoskeletal elongation and limited aggregation. In contrast, cells under aluminum and MCP covers maintained normal morphology and aggregation, suggesting significantly better protection against UV-induced damage. These findings demonstrate that MCP electrospun membranes effectively block UV radiation and help preserve cellular morphology and viability, supporting their potential use in photo-protective biomedical environments.

### 3.6. Antioxidant Assay

Curcumin is known to exert antioxidant effects primarily through free radical scavenging by hydrogen atom donation from its phenolic hydroxyl and methoxy groups [52,53,54]. Additionally, its central methylene group contributes to antioxidant activity via an intramolecular hydrogen shift that facilitates phenolic hydrogen abstraction through alkoxyl radical formation [55]. The antioxidant activities of curcumin, maleic anhydride modified curcumin, and the MCP copolymer were evaluated at a nominal concentration of 1 mg/mL. However, due to the covalent incorporation of curcumin into the MCP polymer backbone, the effective curcumin content in MCP is substantially lower than in free curcumin or MC, and direct comparison should be interpreted with caution. As shown in Table 1, MC exhibited high antioxidant activity, scavenging approximately 88.1% of DPPH radicals within 30 min, followed by curcumin (69.9%) and MCP (41.7%). The reduced antioxidant activity of MCP can be attributed to two factors: (i) the dilution effect from the bioinert PCL segments, and (ii) the consumption of phenolic hydroxyl groups during covalent bond formation in the copolymer, thereby limiting the availability of active hydrogen donors [54]. The superior antioxidant performance of MC suggests that functionalizing phenolic compounds with maleic anhydride may enhance their radical scavenging capacity [56]. However, this improvement may also result from increased solubility or changes in electron distribution, rather than from the MA group alone.

The radical scavenging activity of the MCP-based composite electrospun films was quantified using a solid-phase DPPH assay (Table 1). The base PVA and PVA-MCP formulations exhibited negligible activity (<7%), while metal-loaded variants (Cu or Fe) showed increased antioxidant performance (13.1% and 12.6%, respectively), likely due to redox interactions facilitated by transition metal ions. Although these values are low relative to free curcumin or ascorbic acid, they confirm some capacity for reactive species neutralization under solid-state conditions. Here we acknowledge that while the DPPH scavenging activities of the tested compounds were compared directly with ascorbic acid as a reference, this comparison was largely qualitative rather than strictly quantitative. This is due to differences in molecular weight, concentration, and solvent in the different systems.

### 3.7. Systemic and Contact Toxicity Evaluation of MCP-Based Composite Electrospun Films

**Figure 3:**
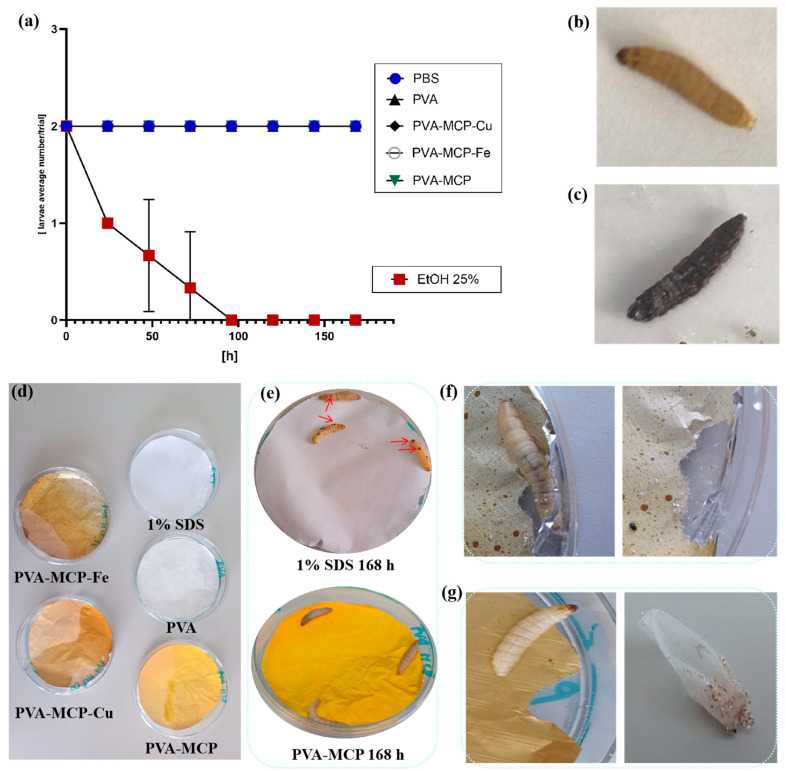
Evaluation of systemic and contact toxicity in *Galleria mellonella* larvae. (**a**) Larvae 168 h post-injection with PBS or eluates from PVA-based spun films (PVA, PVA-MCP, PVA-MCP-Cu, PVA-MCP-Fe) remained viable (**b**), showing no signs of melanization or distress. In contrast, larvae injected with 25% ethanol exhibited complete melanization, loss of turgor, and death within 96 h (**c**). (**d**) Petri dishes lined with the tested spun film samples (PVA, PVA-MCP, PVA-MCP-Cu, PVA-MCP-Fe). (**e**) After 168 h of exposure, larvae exposed to SDS-treated filter paper showed clear signs of cuticular melanization, marked with red arrows, and partial mortality after 168 h. In contrast, PVA-MCP exhibited no signs of melanization or distress after 168 h, confirming the absence of contact toxicity. (**f**,**g**) Larval behavior in contact with test materials, including active gnawing and incorporation of material fragments into cocoon structures, further supports the non-toxic and biocompatible nature of the tested films.

To extend the biological performance of the MCP copolymer, electrospun composite films were fabricated by blending MCP into a PVA matrix, with and without metal additives (Cu, Fe). These films were evaluated for systemic and contact toxicity, and antimicrobial properties. In vivo toxicity was assessed using the *Galleria mellonella* larval model. As shown in Figure 3a–c, no mortality or melanization was observed over 168 h in larvae injected with eluates derived from PVA, PVA-MCP, PVA-MCP-Cu, or PVA-MCP-Fe films. Similarly, no adverse effects were seen in larvae exposed to film surfaces in contact assays (Figure 3d–g), with individuals maintaining normal turgor and mobility. These findings confirm that MCP-based composite films and their metal-containing derivatives are non-toxic and well-tolerated in this preliminary in vivo model.

### 3.8. Evaluation of Antimicrobial Activity and Bacterial Adherence of MCP-Based Composite Electrospun Films

**Figure 4:**
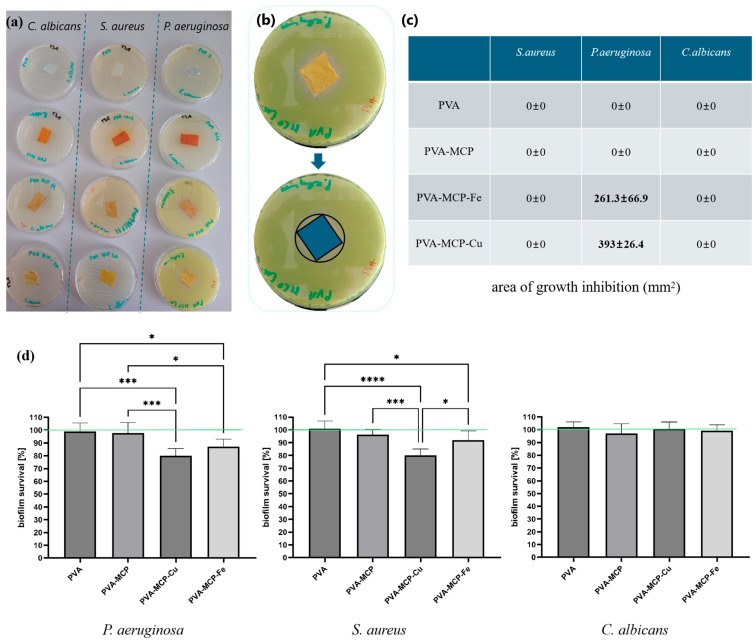
Modified Disk Diffusion assay for evaluating antimicrobial activity in samples and modified Antibiofilm Dressing Activity Measurement (A.D.A.M.) assay. (**a**) Samples were placed on agar surfaces inoculated with tested pathogens. Note: bottom-left PVA-MCP-Cu plates for *S. aureus* and *C. albicans* were inadvertently swapped; this was only a labeling issue in the figure layout and not in the actual experiment. (**b**) Process of calculating area (mm^2^) of growth inhibition. (**c**) Quantitative data showing anti-pseudomonal activity of two types of samples (PVA-MCP-Fe and PVA-MCP-Cu). (**d**) Percentage of biofilm survival after exposure to samples using A.D.A.M. assay. The green horizontal line indicates a level of 100% survival (non-exposed biofilm). Asterisks indicate statistically significant differences between groups (* *p* < 0.05).

**Figure 5:**
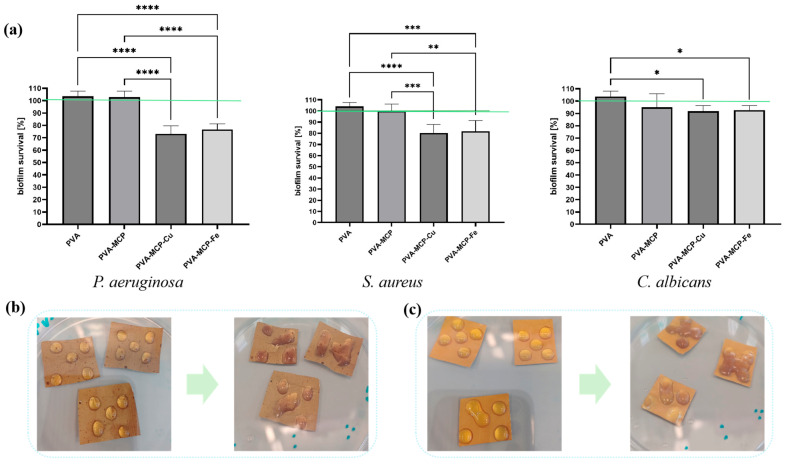
Evaluation of bacterial adherence to sample using a contact TTC reduction assay. (**a**) The green horizontal line indicates a level of 100% survival established for microbials suspended in droplets placed on a non-antimicrobial polystyrene surface. Asterisks indicate statistically significant differences between levels of biofilm survivals (*** *p* < 0.001). (**b**,**c**) Depicted process of the transformation of colorless tetrazolium salt into red formazan as a result of the presence of metabolically active microorganisms suspended in the droplets placed directly on PVA-MCP-Fe or PVA-MCP-Cu, respectively.

Antimicrobial activity was first screened using a modified disk diffusion assay. Only PVA-MCP-Cu and PVA-MCP-Fe generated measurable inhibition zones against *P. aeruginosa*, while no inhibition was observed for *S. aureus* or *C. albicans* (Figure 4a–c). The antibiofilm potential was then quantified using a modified Antibiofilm Dressing Activity Measurement (A.D.A.M.) assay. As shown in Figure 4d, PVA-MCP-Cu and PVA-MCP-Fe significantly reduced *P. aeruginosa* viability and moderately inhibited *S. aureus* biofilms. No significant antifungal effect was observed in these conditions. A surface contact TTC reduction assay revealed reduced microbial adherence and metabolic activity for *P. aeruginosa* and *S. aureus* on the metal-loaded films, confirming their surface-mediated antimicrobial potential. Interestingly, the Fe-containing film also showed a mild reduction in *C. albicans* viability, an effect not previously detected in diffusion-based assays, highlighting the added sensitivity of the contact method (Figure 5).

These results suggest that incorporating MCP into polymeric films enables biocompatibility without systemic toxicity, while the addition of copper or iron ions introduces antimicrobial activity, particularly against Gram-negative bacteria. These findings suggest potential for biomedical applications such as bioactive wound dressings or implantable films, pending further validation.

### 3.9. MCP Scaffold Fabrication

**Figure 6:**
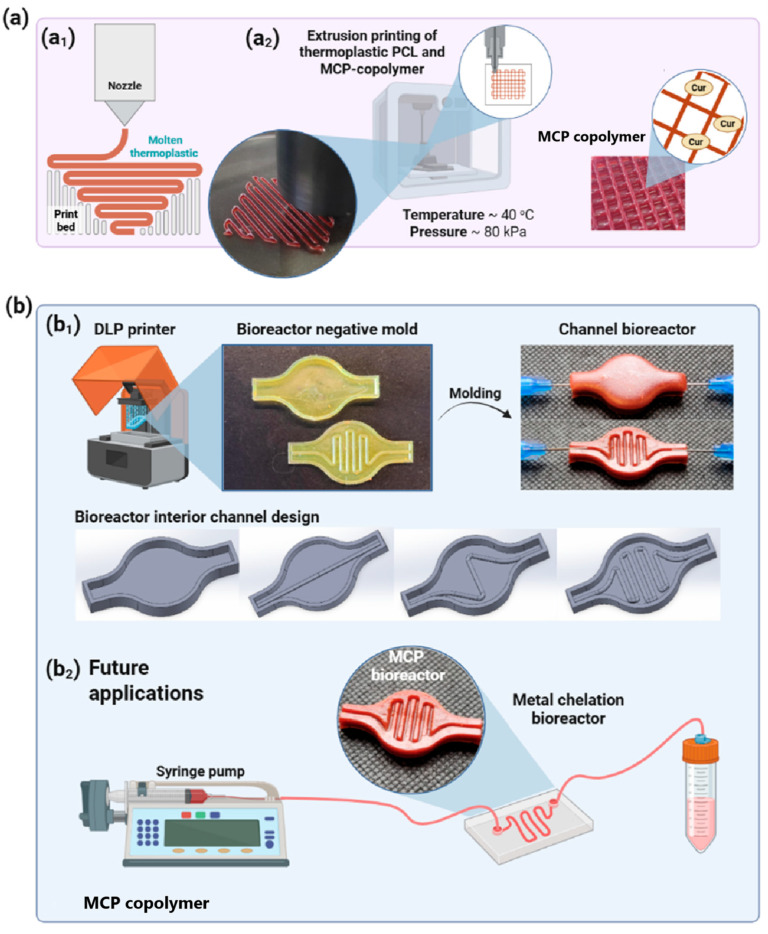
(**a**) Extrusion printing of MCP scaffolds: (**a_1_**) schematic representation of extrusion-based 3D printing using molten thermoplastic MCP copolymer; (**a_2_**) fabricated scaffold with well-defined pore architecture from MCP, printed at ~40 °C and ~80 kPa, demonstrating shape fidelity and uniformity. (**b**) Molding of MCP channel bioreactors: (**b_1_**) design and fabrication of bioreactor molds using DLP 3D printing, followed by molding to produce MCP-based channel bioreactors with customizable internal geometries. (**b_2_**) Prospective applications: the resulting MCP bioreactors are compatible with syringe-pump-driven flow systems for studying flow rate-dependent phenomena and metal chelation kinetics.

The MCP demonstrated processability through various fabrication techniques, including 3D extrusion printing, and molding. These methods enabled the production of scaffolds and molded structures with embedded channels, suitable for diverse biomedical applications. As shown in Figure 6a, 3D-printed scaffolds from MCP exhibited well-organized and uniform pores, with shape fidelity. This underscores the capacity of 3D printing to fabricate MCP scaffolds with customizable shapes and sizes, tailored to specific biomedical needs [57]. Although DSC analysis revealed melting transitions at 53 °C and 61 °C, extrusion was feasible at 40 °C, likely due to softening of the copolymer below Tm, enabling viscous flow under pressure.

In Figure 6b, MCP molding yielded constructs with continuous and well-defined microchannels, enabling controlled fluid flow. These structures can be adapted for applications such as microbial bioreactors, heavy metal adsorption, and hemodialysis systems. Moreover, MCP–Fe complexes could, in principle, act as iron carriers by stabilizing iron in a soluble form and enabling gradual release, with Cu co-loading potentially supporting iron mobilization [58,59]. These applications remain to be validated but highlight the versatility and printability of MCP.

## 4. Conclusions

This study demonstrates the synthesis of a curcumin-functionalized polycaprolactone (MCP) block copolymer via ring-opening polymerization of ε-caprolactone and maleic anhydride modified curcumin. Structural analyses (FTIR, ^1^H NMR, UV–Vis) confirmed the presence of curcumin moieties and their interaction with metal ions. At the same time, DSC showed reduced crystallinity and altered melting transitions compared to neat PCL. Morphological (SEM) and biological assays (cytocompatibility, fibroblast migration, antioxidant, antimicrobial, and in vivo toxicity) collectively demonstrated that curcumin incorporation modified the physicochemical properties of PCL and also imparted bioactivity, enhancing biological performance. Preliminary results demonstrated MCP’s fabrication versatility (electrospinning, 3D printing, molding), showcasing its multifunctional potential for biomedical applications. This work highlights how functionalizing synthetic copolymers with bioactive molecules can yield multifunctional platforms with tunable structural and biological properties.

## Figures and Tables

**Table 1:** The radical scavenging activity of MCP and MCP-based composite electrospun films.

Sample	DPPH Scavenging Activity (%)	SD	Interpretation
**Curcumin**	69.9	2.3	Strong scavenging relative to MCP composites
**MC**	88.1	0.7	Strong scavenging (approaching control)
**MCP**	41.7	0.5	Moderate scavenging activity
**Ascorbic acid**	94.3	1.2	Positive control
**PVA**	4.7	0.5	Negligible scavenging
**PVA-MCP**	6.2	0.4	Negligible scavenging
**PVA-MCP-Cu**	13.1	0.8	Weak scavenging activity
**PVA-MCP-Fe**	12.6	1.0	Weak scavenging activity
**DPPH alone**	0.0	0.0	Negative control

## Data Availability

The raw data supporting the conclusions of this article will be made available by the authors on request.

## Supplementary Materials

The following supporting information can be downloaded at: https://www.mdpi.com/article/10.3390/ma18184348/s1, Figure S1: Ratio of curcumin and polycaprolactone (PCL) in MCP determined by ^1^H NMR intergration; Figure S2: Schematic illustration of the proposed chelation mechanism of curcumin with Cu^2+^ or Fe^3+^ ions in the MCP structure, coordinated via the β-diketone moiety.
