# Supplementary material for: Synthesis and Characterization of Curcumin-Polycaprolactone Block Copolymers for Biomedical Applications
Source: Materials (Basel). 2025 Sep 17;18(18):4348. doi: 10.3390/ma18184348 (PMC12471959; doi:10.3390/ma18184348)
Supplement: Supplementary file 1 [file materials-18-04348-s001.zip › materials-3802520-supplementary.pdf]

Synthesis and Characterization of Curcumin-Polycaprolactone Block Copolymers for Biomedical Applications

Qianqian Wei [1], Adam Junka [2], Bartlomiej Dudek [2], Houman Alimoradi [1], Julia Simińska-Stanny [1], Lei Nie [3,*], Oseweuba Valentine Okoro [1] and Armin Shavandi [1,*]

[1] 3BIO-BioMatter, École Polytechnique de Bruxelles, Université Libre de Bruxelles (ULB), Avenue F.D. Roosevelt, 50-CP 165/61, 1050 Brussels, Belgium
[2] Platform for Unique Models Application PUMA, Department of Pharmaceutical Microbiology and Parasitology, Wroclaw Medical University, Borowska 211, 50-556 Wroclaw, Poland
[3] College of Life Sciences, Xinyang Normal University, Xinyang 464000, China
* Correspondence: nielei@xynu.edu.cn (L.N.); armin.shavandi@ulb.be (A.S.)

NMR integration results indicate that the ratio of PCL to curcumin in MCP is approximately 50:1.

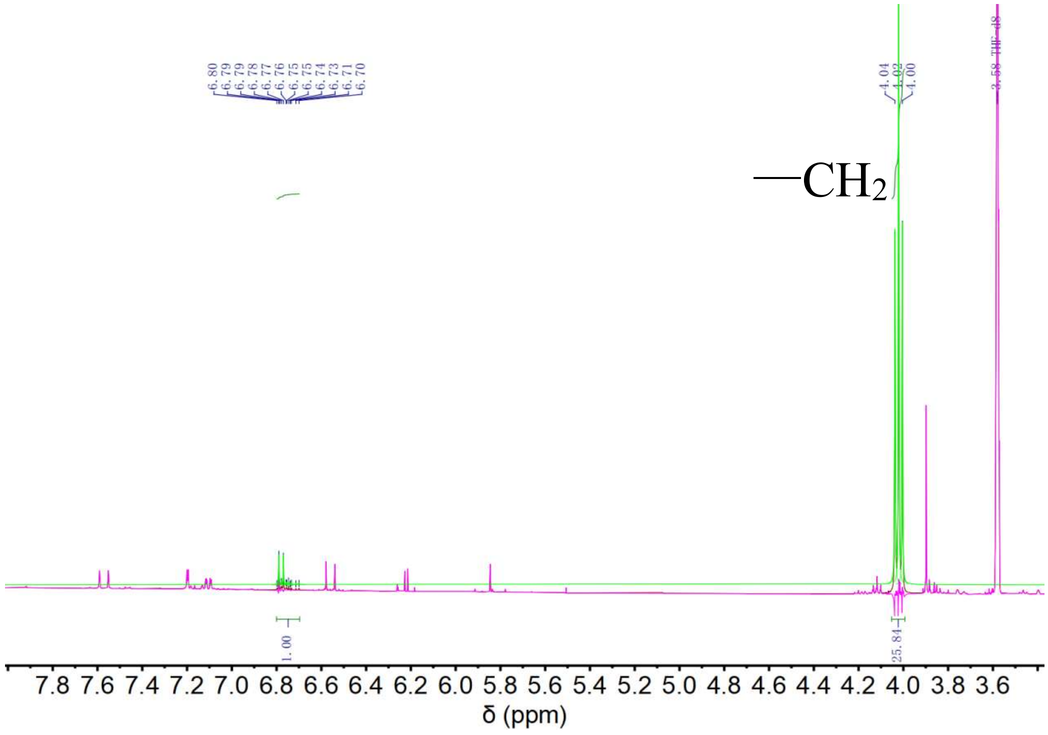

Figure S1 Ratio of curcumin and polylactone (PCL) in MCP determined by ^{1}H NMR intergration.

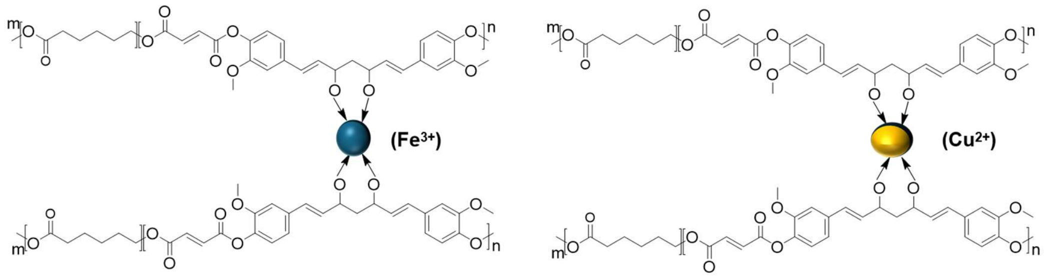

Figure S2 Schematic illustration of the proposed chelation mechanism of curcumin with Cu^{2+} or Fe^{3+} ions in the MCP structure, coordinated via the β-diketone moiety.
